# Early intervention using high-precision radiotherapy preserved visual function for five consecutive patients with optic nerve sheath meningioma

**DOI:** 10.1007/s10147-018-1284-5

**Published:** 2018-04-30

**Authors:** Toshihiko Inoue, Osamu Mimura, Norihisa Masai, Atuyuki Ohashi, Koji Ikenaga, Yoshishige Okuno, Iku Nishiguchi, Ryoongjin Oh

**Affiliations:** 1 Ashiya Radiotherapy Clinic Nozomi, 3-84 Yoko-cho, Ashiya, 659-0034 Japan; 2 Department of Neuro-ophthalmological Therapeutics, Hyogo College of Medicine, Nishinomiya, Japan; 3 Miyakojima IGRT Clinic, Osaka, Japan

**Keywords:** Optic nerve sheath meningioma, IMRT, FSRT, Early intervention, Visual function

## Abstract

**Background:**

There has been a paradigm shift in the treatment for optic nerve sheath meningioma (ONSM) from surgery to fractionated stereotactic radiotherapy (FSRT) in other countries. However, FSRT has seldom been performed in Japan. The purpose of this retrospective study is to reconfirm the effectiveness of early intervention with precision radiotherapy for ONSM reported in our previous study.

**Methods:**

Five consecutive patients with ONSM were retrospectively analyzed. All patients underwent intensity-modulated radiotherapy (IMRT) or FSRT. They received the early interventions between 1.5 and 7 months after deterioration of the disease. The median dose was 52.8 Gy (range 46.0–59.4 Gy) and the median number of fractions was 25 (range 22–33).

**Results:**

All patients experienced reestablishment of vision at the median follow-up time of 36 months (range 18–54 months). Four of them noted early improvement of visual deficits during the treatment course (range 2–4 weeks) and the remaining patient improved 3 weeks after completion of IMRT. The median tumor reduction was 53% (range 39–75%). One patient with diabetes mellitus developed retinal bleeding as a result of radiation retinopathy 16 months after IMRT, although the doses were acceptable. The remaining 4 patients have no late toxicity at the follow-up time of 31–54 months.

**Conclusions:**

A paradigm shift is necessary from surgery to early intervention using precision radiotherapy for the treatment of ONSM in Japan.

## Introduction

There has been a paradigm shift in the treatment of ONSM from surgery to fractionated stereotactic radiotherapy (FSRT) during the last 2 decades in other countries, such as USA and Germany [1, 2]. Optic nerve sheath meningioma (ONSM) is rare tumor and could potentially be treated by a neurosurgeon, plastic surgeon, or ophthalmological surgeon in Japan. In addition, most Japanese surgeons use surgical interventions at an advanced stage of visual loss, i.e., blindness. Accordingly, FSRT has seldom been performed in our country. In a previous case report, we described early intervention with FSRT in a patient with ONSM that resulted in a rapid and complete improvement of visual impairment [3]. As 4 patients, who underwent intensity-modulated radiotherapy (IMRT) in another clinic, have since been added into the study, 5 consecutive patients with ONSM were reviewed and retrospectively analyzed.

## Patients and treatment methods

### Patients

Between September 2013 and May 2015, we accrued 5 consecutive patients with ONSM who underwent IMRT or FSRT for analysis in this study. All patients were diagnosed with ONSM by an ophthalmologist (OM). They were examined by one of the authors (TI), who has consecutively belonged to both clinics, i.e., Miyakojima IGRT Clinic and Ashiya Radiotherapy Clinic Nozomi, to make a treatment decision about IMRT or FSRT. We obtained written informed consent from the patient for publication of the case details and any accompanying images. The plenary meeting of this clinic approved the study design, which included a chart review [3]. With regard to the additional 4 patients treated with IMRT in another clinic, the study protocol with opt-out consent was approved by the Institutional Review Board (IRB). Informed consent was obtained from all 4 patients.

Patients and tumor characteristics are shown in Table 1. The median age was 46 years (range: 34–61 years). There were 2 men and 3 women. One of the patients had intercurrent diabetes mellitus. Three patients had lesions in their left eye and 2 had lesions in their right eye. Four patients had tumors located in the intraorbital region. The remaining patient had a tumor in the intracanalicular region. Concerning the shape of tumors, there were fusiform and tubular tumor types in 2 patients each, and a global tumor in the remaining patient [4]. The median volume of the tumor was 0.44 cm^3^ (range 0.20–0.90 cm^3^). At the time of treatment initiation, the median corrected visual acuity (VA) in the involved eye was 0.29 (log MAR visual acuity: 0.544) (range 0–1.7). We set MAR to the minimum angle of resolution. The median value of the central critical flicker-fusion frequency (CFF), which represents functional impairment of the optic nerve before the significant depression of VA, was 23.5 (range N/V–41.5). With regard to the visual field (VF), 3 patients suffered from quadrantanopsia and two had centrocaecal scotoma. The mean deviation (MD) of sensitivity depression was − 16.18 (range − 1.30 to N/A) in the involved eye with Humphrey automated perimetry (HAP).

**Table 1:** Patient and tumor characteristics

#Patient	#1	#2	#3	#4	#5
Age (years)	35	57	46	61	41
Sex	Female	Female	Female	Male	Male
Intercurrent disease	(−)	(−)	(−)	Diabetes mellitus	(−)
Tumor					
Laterality	Left	Right	Left	Right	Left
Location	Intraorbital	Intraorbital	Intracanalicular	Intraorbital	Intraorbital
Shape	Fusiform	Fusiform	Tubular	Tubular	Globular
Size (mm)	23 × 9 × 10	13 × 5 × 6	16 × 10 × 8	18 × 9 × 8	9 × 6 × 8
Volume (cm^3^)	0.87	0.20	0.44	0.90	0.30
Corrected VA in the affected eye					
Log MAR (decimal)	0.3 (0.5)	0.22 (0.6)	0.5 (0.3)	0 (1.0)	1.7 (0.02)
CFF					
Right (Hz)	43–44 (43.5)	30	53/42 (47.5)	41/42 (41.5)	41/42 (41.8)
Left (Hz)	23–24 (23.5)	40	13/10 (11.5)	38/41 (39.5)	N/A
Visual field	Quadrantanopsia	Quadrantanopsia	Centrocecal scotoma	Quadrantanopsia	Centrocecal scotoma
HAP					
Right MD (dB)	− 0.59	− 16.18 *p* < 0.5%	− 1.36 *p* < 10%	− 1.30	N/A
Left MD (dB)	− 8.9	− 0.12	− 24.35 *p* < 0.5%	+ 2.00	N/A

*VA* visual acuity, *LogMAR* logarithm of minimum angle of resolution, *CFF* critical flicker-fusion frequency, *N/A* not available, *HAP* Humphrey automated perimetry, *MD* mean deviation of sensitivity depression with HAP

### Methods

Treatment characteristics are summarized in Table 2. The median time from disease onset and rapid deterioration to treatment initiation was 19 and 3 months (range 11–41 and 1.5–7 months), respectively. Four patients underwent IMRT and the remaining patients received FSRT. The median prescription dose was 52.8 Gy (range 46.0–59.4 Gy). The median number of fractions was 25 (range 22–33). The median overall treatment days were 35 days (range 31–45 days). Median numbers of beams was 7 (range 5–9). All patients were treated with a 6 MV X-ray Novalis unit™ (BrainLAB AG, Munich, Germany).

**Table 2:** Treatment characteristics

#Patient	#1	#2	#3	#4	#5
Onset to Trx (months)	41	19	19	20	11
Deterioration to Trx (months)	7	3	2	4	1.5
Trx method	IMRT	IMRT	IMRT	IMRT	FSRT
Prescription dose (Gy)	59.4	54	52.8	46	50
#Fractions	33	30	22	23	25
Days	45	43	33	31	35
#Beams	7 non-coplanar	7 non-coplanar	9 non-coplanar	7 non-coplanar	5 coplanar

*Trx* treatment, *IMRT* intensity-modulated radiotherapy, *FSRT* fractionated stereotactic radiotherapy, *NED* no evidence of disease

In general, as the size of the target volume increases, the risk of damage to the organ at risk (OAR) such as the optic nerve, chiasm, and retina increases. Therefore, for four patients treated with IMRT, we tried to decrease the planning target volume (PTV) as small as possible. The gross tumor volume (GTV) was determined with CT-MRI fusion image to improve the accuracy of delineation of the GTV. We defined the GTV as the clinical target volume (CTV). In the parts with mobility accompanying eyeball movements, the internal margin (IM) of 1 mm was added to the CTV in every direction excluding anterior and was defined as internal target volume (ITV). In other parts, we defined the CTV as the ITV. The PTV was defined as the ITV plus the set-up margin (SM) of 2 mm. Therefore, the GTV mean of 0.6 cm^3^ resulted in the PTV mean of 2.2 cm^3^. In dose constraints for OARs, we determined *D*_2%_ and *D*_mean_ of the optic nerve, chiasm, and retina as upper limits of biologically equivalent dose at 2 Gy per fraction (EQD_2_) of 60 Gy and 45 Gy, respectively. In the case of patient #5 treated with FSRT, we defined GTV of 0.3 cm^3^ using CT and MRI, and the PTV was 1.7 cm^3^, which was based on a 3-mm margin added to the ITV [3]. Details of the treatment devices and methods were reported previously [3, 5, 6].

## Results

All patients had improved and re-established VA and VFs at the median follow-up time of 36 months (range 18–54 months). Four of the patients experienced improvement in their visual deficits during the treatment course (range 2–4 weeks). The remaining patient showed improvement 3 weeks after completion of IMRT (Table 3). Since the tumors showed slow regression after completion of IMRT or FSRT based on the benign nature of the disease, the median reduction of the tumor size was 53% (range 39–75%). One patient achieved a 53% tumor reduction at 16 months; however, he was lost to follow-up 18 months after the completion of radiotherapy. The remaining 4 patients have not experienced any adverse events, and have returned to their normal lives.

**Table 3:** Treatment results

#Patient	#1	#2	#3	#4	#5
Early improvement of					
Visual acuity (weeks)*	Yes (9)	Yes (4)	Yes (4)	Yes (2)	Yes (2)
Visual field (weeks)*	Yes (9)	Yes (4)	Yes (2)	Yes (2)	Yes (2)
Final reestablishment in involved eye					
Visual acuity (months)*	(1.2) (51)	(1.2) (43)	(1.2) (34)	(0.9) (18)	(1.2) (27)
MD (dB) (months)*	− 0.32 (51)	− 0.81 (43)	− 3.54 (34)	N/A (18)	+ 0.61 (27)
Tumor response					
Tumor size (mm)	16 × 8 × 6	11 × 4 × 5	11 × 8 × 6	16 × 6 × 6	5 × 3 × 6
Tumor volume (cm^3^)	0.47	0.12	0.11	0.42	0.08
Tumor reduction (%) (months)*	46% (54)	39% (46)	75% (36)	53% (18)^$^	73% (27)
Status	NED	NED	NED	Retinal bleeding	NED
Follow-up (months)*	54	46	36	18	34

*NED* no evidence of disease, *MD* mean deviation of sensitivity depression with Humphrey automated perimetry

*After the start of IMRT or FSRT

^$^At the last examination, thereafter lost to follow-up

The dose-volume histogram (DVH) parameters for the planning target volume (PTV), gross tumor volume (GTV), optic nerve, retina, disk, eyeball and chiasm of the 5 patients are listed in Table 4. The median *D*_95%_, i.e., minimum dose in 95% of the PTV was 47.9 Gy (range 45.1–58.9 Gy), and median *D*_98%_, i.e., near-minimum dose of GTV was 51.7 Gy (range 46.7–60.7 Gy). The median *D*_2%_, i.e., near-maximum dose of the optic nerve and retina was 55.0 Gy (range 47.8–63.2 Gy) and 8.0 Gy (range 0.9–47.2 Gy), respectively.

**Table 4:** DVH parameters

#Patient (prescription dose)	#1 (59.4 Gy)	#2 (54 Gy)	#3 (52.8 Gy)	#4 (46 Gy)	#5 (50 Gy)
PTV					
*D*_95%_	58.9 Gy	50.3 Gy	45.8 Gy	45.1 Gy	47.9 Gy
*V*_107%_	0.1 cm^3^	0 cm^3^	0.3 cm^3^	0 cm^3^	0.6 cm^3^
*D*_98%_	58.0 Gy	45.6 Gy	43.5 Gy	44.7 Gy	47.0 Gy
*D*_50%_	61.6 Gy	54.4 Gy	53.5 Gy	46.9 Gy	52.5 Gy
*D*_2%_	63.3 Gy	56.1 Gy	56.3 Gy	47.8 Gy	55.5 Gy
GTV					
*D*_98%_	60.7 Gy	54.5 Gy	50.4 Gy	46.7 Gy	51.7 Gy
*D*_50%_	61.8 Gy	55.2 Gy	54.5 Gy	47.3 Gy	54.4 Gy
*D*_2%_	63.3 Gy	56.0 Gy	56.4 Gy	47.8 Gy	55.6 Gy
Optic nerve					
*D*_2%_	63.2 Gy	56.0 Gy	55.0 Gy	47.8 Gy	54.0 Gy
${D_{0.1c{m^3}}}$	62.5 Gy	55.5 Gy	40.1 Gy	47.7 Gy	52.2 Gy
*D*_mean_	52.3 Gy	38.1 Gy	12.6 Gy	31.5 Gy	25.8 Gy
Retina					
*D*_2%_	19.5 Gy	8.0 Gy	0.9 Gy	47.2 Gy	1.6 Gy
${D_{0.1c{m^3}}}$	16.3 Gy	6.9 Gy	0.6 Gy	46.0 Gy	1.2 Gy
${D_{0.5c{m^3}}}$	4.0 Gy	2.1 Gy	0.4 Gy	17.3 Gy	0.7 Gy
${D_{1c{m^3}}}$	1.1 Gy	0.6 Gy	0.3 Gy	0.2 Gy	0.3 Gy
*D*_mean_	2.8 Gy	2.0 Gy	0.3 Gy	13.0 Gy	0.8 Gy
Disk (point dose)	23.4 Gy	8.0 Gy	0.5 Gy	47.2 Gy	5.0 Gy
Eyeball					
*D*_2%_	12.0 Gy	6.7 Gy	0.5 Gy	45.0 Gy	1.2 Gy
${D_{1c{m^3}}}$	4.4 Gy	2.1 Gy	0.5 Gy	24.9 Gy	0.7 Gy
${D_{5c{m^3}}}$	0.7 Gy	0.3 Gy	0.3 Gy	1.2 Gy	0.3 Gy
*D*_mean_	1.5 Gy	1.2 Gy	0.3 Gy	8.8 Gy	0.4 Gy
Chiasm					
*D*_2%_	2.6 Gy	1.9 Gy	55.0 Gy	0.5 Gy	19.2 Gy
${D_{0.1c{m^3}}}$	1.1 Gy	0.9 Gy	21.3 Gy	0.3 Gy	15.1 Gy
*D*_mean_	0.9 Gy	0.5 Gy	17.3 Gy	0.2 Gy	7.5 Gy

*DVH* dose-volume histogram, *PTV* planning target volume, *GTV* gross tumor volume, *Dv* absorbed dose in fraction *V*_*%*_ (or *V* cm^3^) of the volume in the organ, *V*_*D*_ volume receiving at least an absorbed dose *D*% (or *D* Gy), *D*_*mean*_ mean absorbed dose

**Fig. 1:**
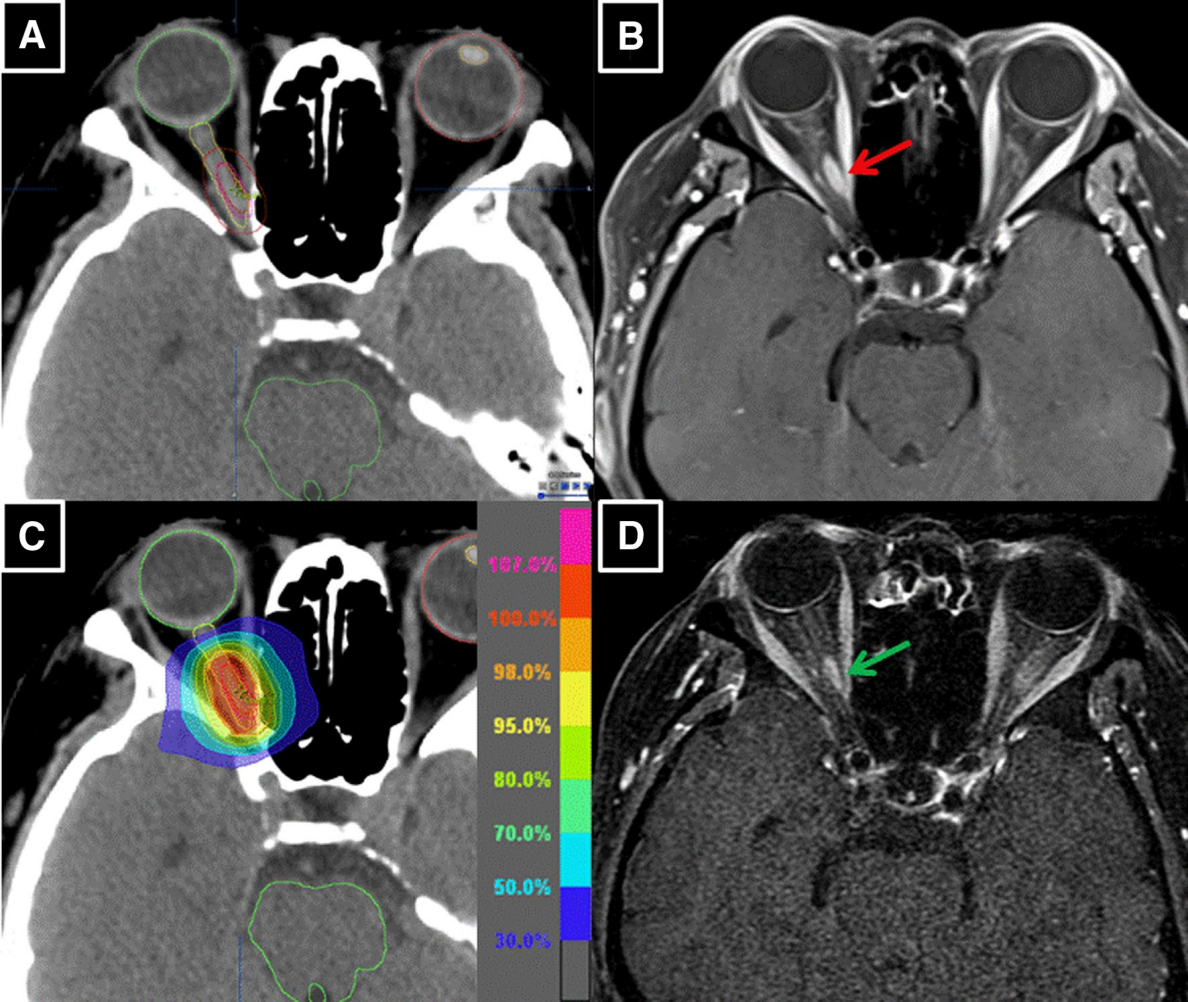
Patient #2 was a 57-year-old woman. Pretreatment computed tomography (CT) (**a**) and magnetic resonance imaging (MRI) showed a 13-mm-long, fusiform type of intraorbital tumor (red arrow) on the medial side of right optic nerve (**b**). She underwent IMRT of 54 Gy in 30 fractions over 43 days in January 2014 (**c**). MRI performed 46 months after IMRT revealed 39% reduction of the tumor volume (green arrow) (**d**)

**Fig. 2:**
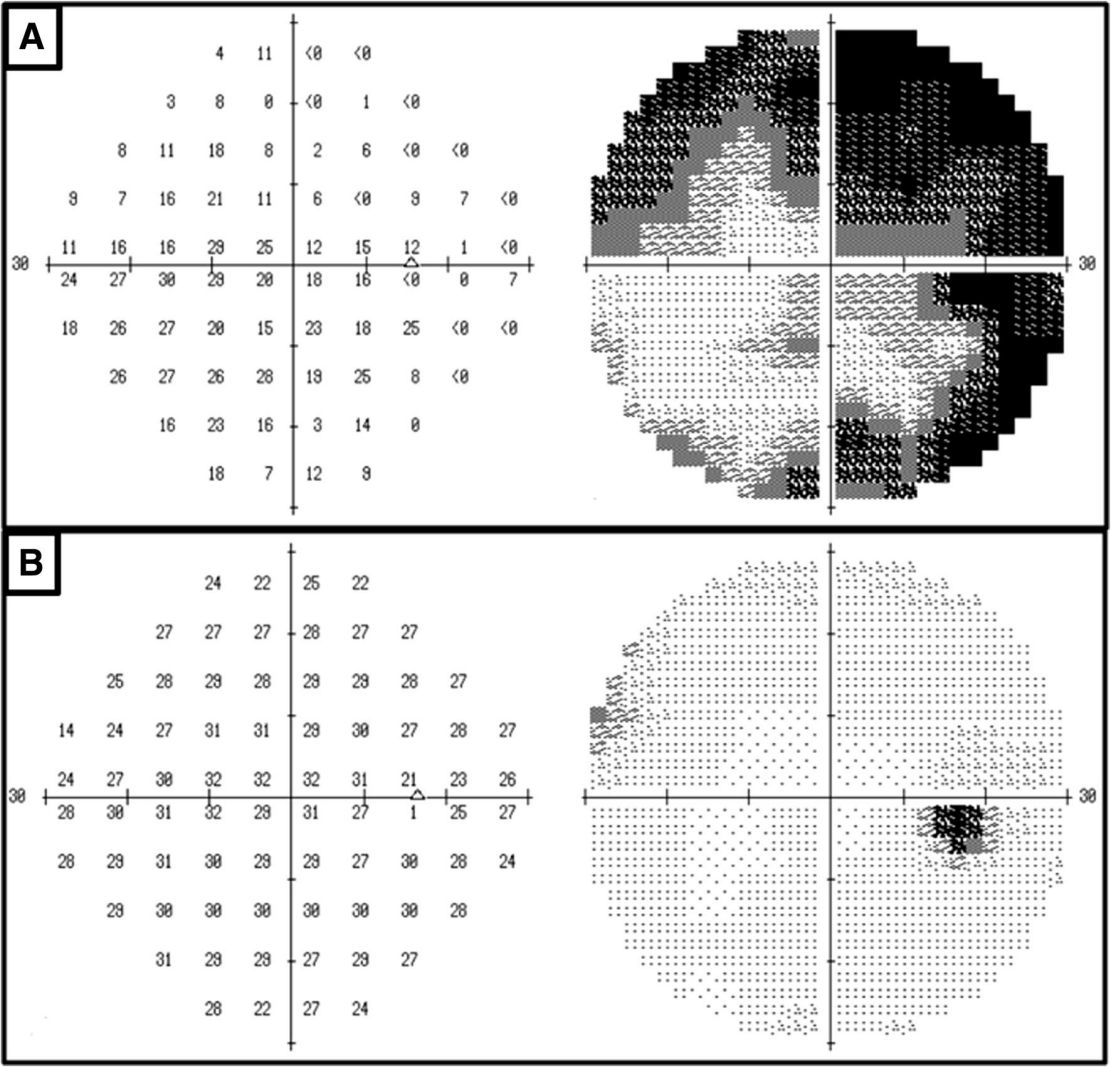
Patient #2 had quadrantanopsia before treatment initiation on December 26, 2013. Humphrey automated perimetry (HAP) showed MD = − 16.18 (**a**). During the course of 4 weeks, HAP showed near-complete disappearance of the visual field deficit with MD = − 1.02 dB on February 27, 2014 (**b**)

Patient #2 received a prescription dose of 54 Gy (Fig. 1; Table 2). An early improvement of VA and VFs was observed during the treatment course of 4 weeks (Fig. 2; Table 3). *D*_95%_ of the PTV, *D*_98%_ of the GTV, and *D*_2%_ of the optic nerve were 50.3, 54.5, and 56.0 Gy, respectively. However, *D*_2%_ and *D*_0.1cm_^3^ of the retina in the involved eye were 8.0 and 6.9 Gy, respectively (Table 4), which remained within the acceptable dose range to avoid radiation retinopathy. The patient survives without any evidence of disease and has good visual function, i.e., log MAR = − 0.08/− 0.08 (VA = 1.2/1.2) and median depression (MD) of HAP = − 0.55/− 0.32 (Fig. 2).

**Fig. 3:**
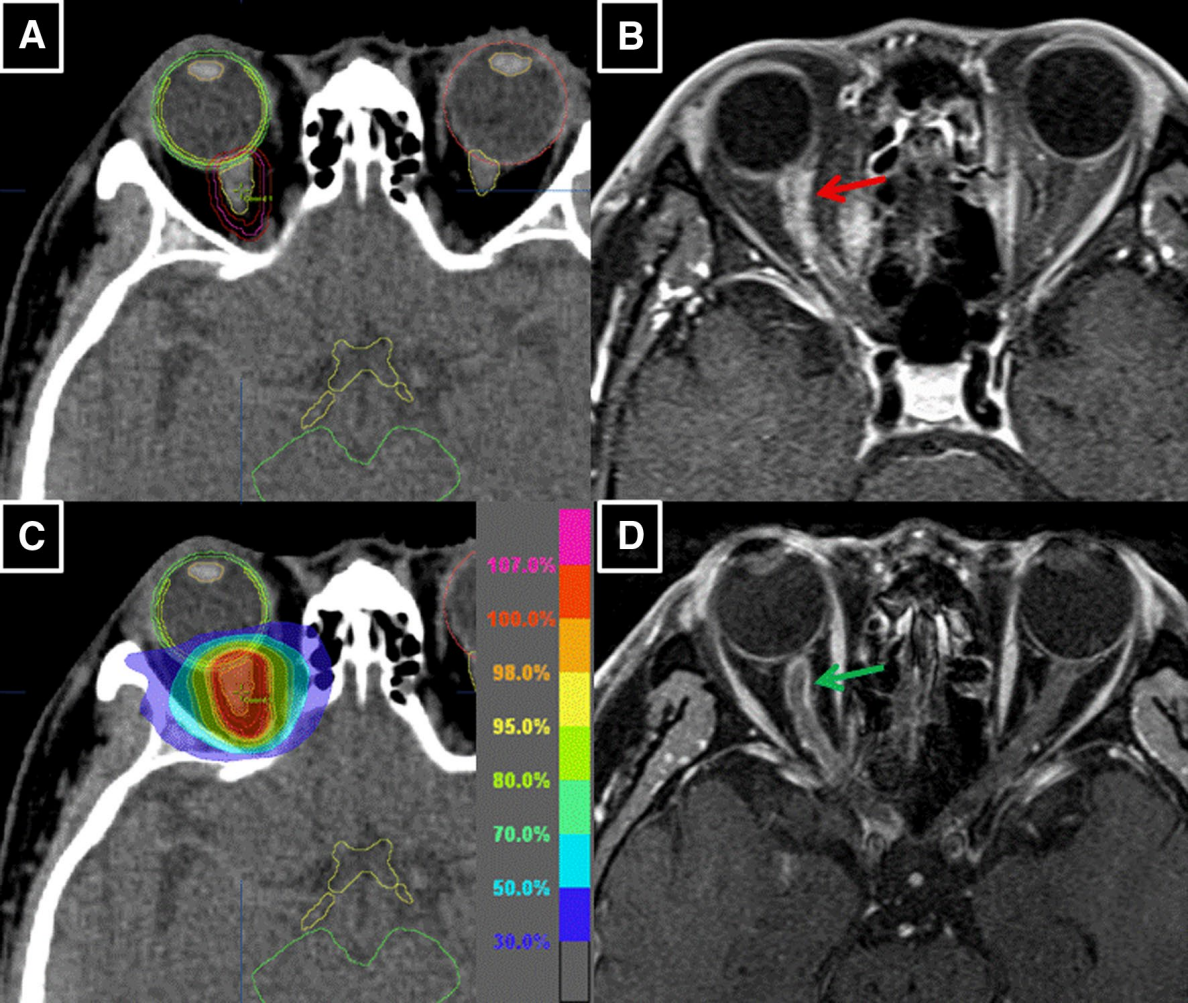
Patient #4 was a 61-year-old man. Pretreatment CT (**a**) and MRI showed a tubular intraorbital tumor (red arrow) of 8 × 3 mm located on the proximal portion of the right optic nerve. A “tram-track” sign was also detected (**b**). He underwent IMRT of 46 Gy in 23 fractions over 31 days in February 2015 (**c**). MRI taken in August 2015 revealed 53% reduction of the tumor volume (green arrow) (**d**)

**Fig. 4:**
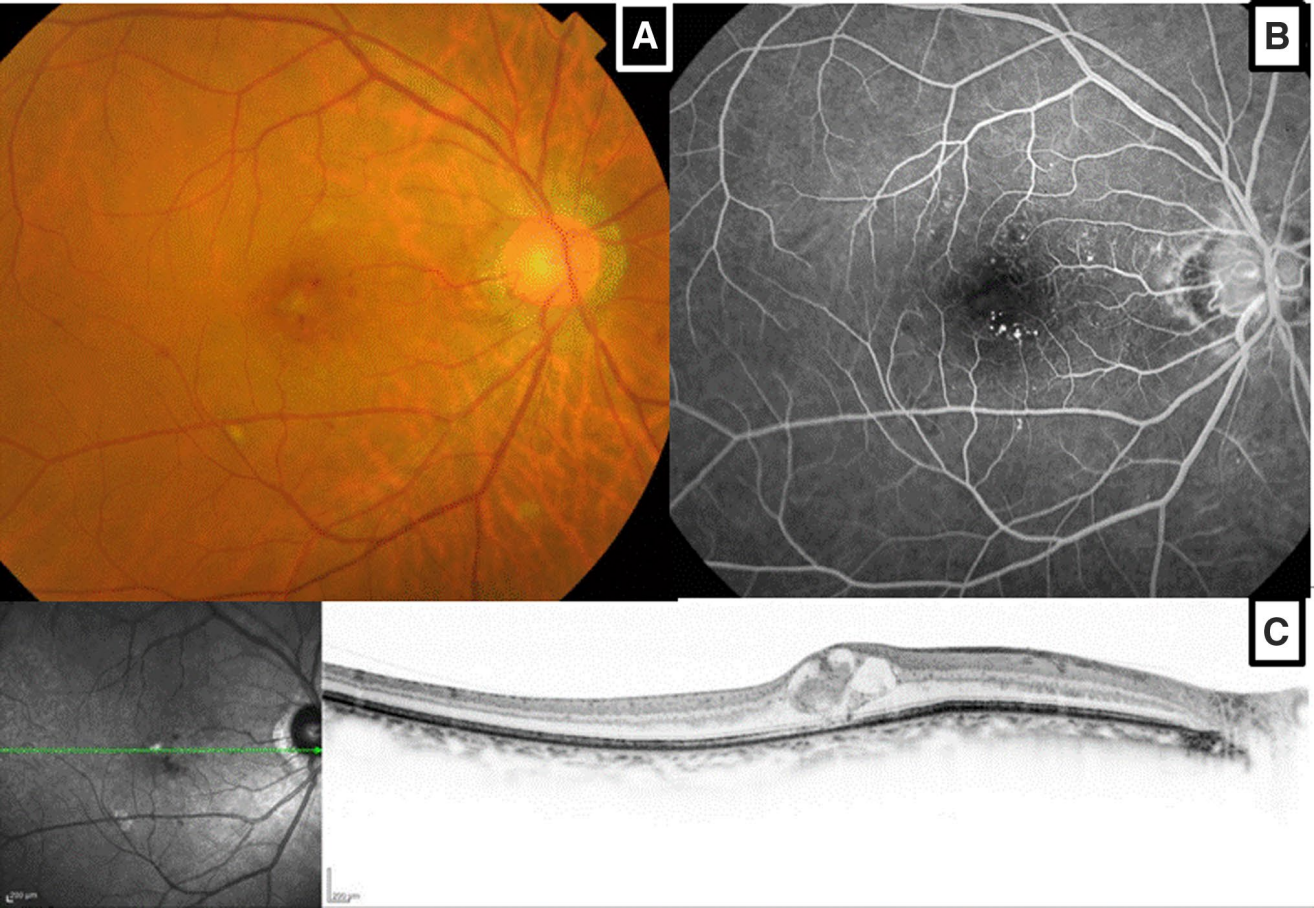
Patient #4 with intercurrent diabetes mellitus developed radiation retinopathy. A color fundus photograph showed retinal bleeding at the right macula, which developed 16 months later after completion of IMRT (**a**). Microaneurysms were observed on the right macula 30.1 s after intravenous injection of fluorescent angiography (**b**). Optical coherence tomography (OCT) showed cystoid macular edema in the horizontal plane (**c**)

In contrast, in patient #4, the final dose reduced to 46 Gy from the initial planning dose of 50 Gy because of his intercurrent diabetes mellitus (Fig. 3; Table 2). Early improvement in his vision was also observed during the treatment course of 2 weeks (Table 3). *D*_2%_ and ${D_{0.1c{m^3}}}$ of the retina in the involved eye were 47.2 and 46.0 Gy, respectively, because of the location of his tumor on the proximal portion of the optic nerve (Fig. 3; Table 4). Despite the acceptable dose levels proposed with QUANTEC (The Quantitative Analysis of Normal Tissue Effects in the Clinic) [7], the patient developed retinal bleeding from the radiation retinopathy 16 months after IMRT (Fig. 4). The combined effect of the posterior retina dose and his intercurrent diabetes mellitus may have caused his retinal bleeding.

## Discussion

Because of the extremely rare incidence of this disease, it is sometimes difficult to diagnose as primary ONSM. ONSMs comprise 2% of all orbital tumors and 1–2% of all meningiomas [8]. Furthermore, primary ONSM represents only 10% of all ONSM, all other (90%) being the secondary ONSM. In our previous report [3], patient #5 was initially diagnosed as Leber hereditary optic atrophy by the first ophthalmologist, because of the progressive visual loss with central scotoma. An ophthalmologist (O.M.) diagnosed this condition as intraocular lesion, which resulted in the compression of left optic nerve (ON) because of the absence of the family history and the presence of decreased light reflex. MRI showed a globular tumor on the left distal ON at precanalicular portion. Patient was consequently diagnosed with primary ONSM. Many kinds of tumors such as glioma, neurofibroma, schwannoma, fibrous histiocytoma, cavernous hemangioma and so on are the mimicking ONSM. Due to the considerable morbidities associated with biopsy, diagnosis of ONS tumors falls heavily on imaging findings, in addition to the clinical presentation. Unenhanced CT may demonstrate diffuse calcification within or along an ONS complex mass, which are highly suggestive of an ONSM. Gadolinium-based contrast enhanced fat-suppression T1W pulse sequences have made a significant contribution to the orbital imaging and gold standard for evaluation of the optic nerve disorders [9].

In this study, early improvement of VA and VFs was observed during the treatment courses among all 5 patients. However, many investigators have already recognized this phenomenon in other countries [1, 10–12]. Most studies have reported that the underlying mechanism was not clear; however, we suggest that the early decompression of the optic nerve sheath, which resulted from the small reduction in the tumor size in the early stage of FSRT, was one of the main mechanisms. Jeremić et al. also supposed that the main reason for this observation might actually be a combination of a radiation-induced edema decrease and decompression of the functional nerve structures [1].

In the above FSRT studies, it was generally agreed that functional improvements were not followed by a significant decrease in the tumor size on imaging. Accordingly, Maclean et al. concluded that thorough ophthalmologic assessment was important because clinical responses often occurred in the absence of radiological change, based on their experience of meningiomas (including 3 ONSM) causing visual deficits [13]. Moreover, an improvement in visual function could occur even during the course of FSRT, as well as later in the follow-up period. However, there were controversial reports as to the extent of the tumor shrinkage observed after FSRT among large number of the patients with skull base meningioma [14, 15]. Becker et al. reported their experience of 39 patients with ONSM. All patients responded to the treatment. However, almost all patients experienced stable disease, i.e., no change on CT/MRI, and only 1 experienced a partial response [16]. Other investigators reported the same results in the same period [12, 17].

In this series, 1 patient developed retinal bleeding from the radiation retinopathy at 16 months after IMRT, probably because of a combination effect with the posterior retina dose and intercurrent diabetes mellitus. Baumert et al. reported that 1 patient developed radiation retinopathy complicated by a vitreous hemorrhage and cataract 4 years after FSRT [6]. A study by Sitathanee et al. described 12 patients treated with FSRT who received a mean dose of 55.5 Gy (range 51.6–59.1 Gy) delivered in 1.8 Gy daily; 1 patient with uncontrolled diabetes and hypertension developed vitreous hemorrhage 2 years after FSRT [17]. A previous case report described a patient with radiation retinopathy occurring 2 years after FSRT (optic nerve dose, 54 Gy in 30 fractions; optic nerve head dose, 48–54 Gy; posterior retina dose, 27.8–48 Gy) without pretreatment factors contributing to its occurrence. Therefore, it remains unclear why this patient developed retinopathy [18].

There is a great discrepancy between the effective IMRT and/or FSRT dose producing early improvement (20–30 Gy) and definitive control of the disease (50 Gy). The lowest controlled dose might be estimated as 30–40 Gy. If a tumor were controlled with a lower dose, it would be better for patients who have a severe intercurrent disease and a high risk of adverse effects, such as radiation retinopathy and/or optic neuropathy. High-risk patients could refuse definitive radiotherapy for these reasons. However, such high-risk patients cannot undergo radical surgery either. Accordingly, it is important to investigate safer ways to perform precision IMRT or FSRT for these high-risk patients with severe intercurrent disease.

In this retrospective study, no patients developed radiation-induced optic neuropathy during the follow-up time from 18 to 54 months. Median *D*_2%_ and ${D_{0.1c{m^3}}}$ for optic nerve was 55.0 Gy (range 47.8–63.2 Gy) and 52.2 Gy (range 40.1–62.5 Gy), respectively.

Many previous studies have investigated radiation tolerance of the optic nerve. Without previous surgical damage to the optic pathways, with a single dose of < 2.0 Gy and a total dose ranging from 45 to 50 Gy, the risk of radiation optic neuropathy was below 2% [19, 20]. When the total dose increased to 54 Gy, the risk of optic neuropathy rose to 5% [21, 22]. However, in a study of head and neck cancer with a dose of < 59 Gy no injury was observed in 106 optic nerves. With a dose of ≥ 60Gy, the 15-year actuarial risk of developing optic neuropathy was 47% with a fraction size of ≥ 1.9 Gy, but this was only 11% with fractions < 1.9 Gy [2]. Zenda et al. reported that 4 head and neck tumor patients who received a proton dose from 53.2 GyE (Gy equivalent) to 65.3 GyE developed optic nerve disorders [23]. In a study of carbon therapy, 58% patients who received irradiation to the optic nerve with > 57 GyE (*D*_max_) developed radiation optic neuropathy. In addition, a dose of 20% of the volume of the optic nerve (*D*_20_) was significantly associated with visual loss [24].

Because the conventional tolerance dose proposed for the retina and optic nerve estimated from conventional fractionated radiotherapy does not apply to modern precision radiotherapy [7, 25], updated information is necessary to determine the relevant tolerance dose for advanced treatment strategies.

At this moment, there are three primary treatment options for patients with cavernous sinus meningiomas: observation, microsurgical resection, and stereotactic radiosurgery (SRS). SRS may complement surgery or can be only reserved for growing remnants [26]. SRS can be replaced with FSRT or IMRT fundamentally and safely. Advanced radiotherapy techniques with standard fractionation schedules (56 Gy/28 fractions) can be a good option. The reported local control reached 82–95% at 5 years and a radiological response rate of 29–31% was observed. Multisession radiosurgery (mRS) has been evaluated in meningiomas with CyberKnife. When a 25 Gy in 5 fractions scheme is adopted, the total dose is theoretically comparable to the doses delivered with conventional fractionated regimens (50.4–56 Gy). The indication for mRS was given by the proximity to optic nerve and chiasm or the dimension of the lesion (> 3 cm). The local control rate at 5 years was of 95%. Only 3.5% of patients experienced a deterioration of preexisting symptoms. Proton has been also advocated to obtain reduced complication in this specific site. High overall 5-year local control rate (96%) was observed using 57EGy without major toxicity in Loma Linda University [27].

Early intervention with IMRT as well as FSRT resulted in a rapid improvement and reestablishment of visual impairment in 5 patients with ONSM. One patient with diabetes mellitus developed radiation retinopathy 16 months after completion of IMRT. The remaining 4 patients have not experienced any late toxicity at the follow-up time from 34 to 54 months. Accordingly, we suggest that early intervention with IMRT as well as FSRT is an effective and standard treatment option for not only stabilizing but also improving the vision of patients with primary ONSM. There has been a substantial shift in the treatment for ONSM from surgery to FSRT in US and EU. While a comprehensive evaluation of radiotherapy is not yet at hand, due to the benign nature of ONSM, limited number of this disease and thus decades of follow-up needed, it seems that this treatment option with early intervention using IMRT and FSRT might in future represent the mainstay of therapy for ONSM in Japan.
